# Type 1 diabetes induces biomechanical changes in skeletal muscle of Wistar rats?

**DOI:** 10.1186/1758-5996-7-S1-A7

**Published:** 2015-11-11

**Authors:** Camilla Rodrigues de Souza Silva, Marcos Paulo Galdino Coutinho, Marcos Vinícius Falcão Amaral, Bruna Soares Teixeira de Araujo, Ana Camila Nobre de Lacerda Brito, Patrícia Verçoza de Castro Silveira, Diogo Arruda Martins de Lima, Magno Felipe Holanda Barboza Inácio Teixeira, Márcio Almeida Bezerra, Silvia Regina Arruda de Morae

**Affiliations:** 1 Universidade Federal de Pernambuco, Recife, Brazil

## Background

Chronic hyperglycemia caused by diabetes mellitus type 1 is associated with damage, dysfunction and failure of several organs and systems, including the musculoskeletal system[1]. In the installed framework of diabetic insulin deficiency, there is an imbalance between the rates of protein synthesis and degradation, causing a condition called diabetic myopathy[2]. However, there are no reports about investigations of biomechanical characteristics of skeletal muscles in diabetic state.

## Objectives

To evaluate the biomechanical properties of the gastrocnemius muscles of rats induced to experimental type 1 diabetes.

## Materials and methods

Male Wistar rats were used and were divided into two groups: a) control group, CG (n=11); b) Diabetic Group, GD (n=19). The GD group was induced to diabetes by intraperitoneal administration of Streptozotocin. After nine weeks, the gastrocnemius-plantar complex of all groups was collected and forwarded to the mechanical tests, which provided the biomechanical parameters. For statistical analysis Kolmolgorov-Smirnov normality test was used, with the Student t test for parametric data and Mann-Whitney test for nonparametric, p <0.05.

## Results

Biomechanical testing GD group exhibited lower values for the variables: maximum power (GC 51.5±9.21, 26.52±9.74 GD; p=0.0001), deformation (GC 17.85±5 75; GD 10.2±3.30; p=0.002), specific strain (GC 41.04±15.89, GD 26.59±8.23, p=0.019), force/width (CG 2 06±0.37, 1.05±0.39 GD; p <0.001) power/area (GC 45.58±2.71; GD 4.9±2.21; p=0.003), cross-sectional area (GC 67.49±16.37; 30.13±7.48 GD; p=0.0001). There was no difference in voltage values at full strength and elastic modulus. In clinical and metabolic parameters, experimental diabetes reduced body weight (GC 429.8±32,65g; GD 238.8±26,98g, p <0.001) and increased blood glucose values when compared to GC group from the first week post induction by the end of the experiment (GC 103.82±16.59/GD 471.30±71.55, p <0.001).

## Conclusion

Most of the evaluated parameters showed a biomechanical disadvantage in the gastrocnemius-plantar complex of animals submitted to Diabetes type 1, making them weaker when subjected to traction.
